# Biodiscovery of Potential Antibacterial Diagnostic Metabolites from the Endolichenic Fungus *Xylaria venustula* Using LC–MS-Based Metabolomics

**DOI:** 10.3390/biology10030191

**Published:** 2021-03-04

**Authors:** Krystle Angelique A. Santiago, RuAngelie Edrada-Ebel, Thomas Edison E. dela Cruz, Yuen Lin Cheow, Adeline Su Yien Ting

**Affiliations:** 1 School of Science, Monash University Malaysia, Jalan Lagoon Selatan, Bandar Sunway, Selangor Dahrul Ehsan 47500, Malaysia; krystle.santiago@monash.edu (K.A.A.S.); cheow.yuen.lin@monash.edu (Y.L.C.); 2 Tropical Medicine & Biology Multidisciplinary Platform, Monash University Malaysia, Jalan Lagoon Selatan, Bandar Sunway, Selangor Dahrul Ehsan 47500, Malaysia; 3 Strathclyde Institute of Pharmacy and Biomedical Sciences, University of Strathclyde, The John Arbuthnott Building, 161 Cathedral Street, Glasgow G4 0RE, UK; ruangelie.edrada-ebel@strath.ac.uk; 4 Department of Biological Sciences, College of Science, University of Santo Tomas, España Boulevard, Manila 1008, Philippines; tedelacruz@ust.edu.ph

**Keywords:** dereplication, endolichenic, LC–MS, metabolomics, OPLS-DA, PCA, PLS-DA, *Xylaria*

## Abstract

**Simple Summary:**

In this study, we determined the bioactivities and chemical natures of three species of lichen *Usnea* and their associated endolichenic fungi (ELF) through metabolomics. We found significant differences in the antibacterial activities and the metabolites produced by the host lichen and its ELF, with the latter targeting a wider scope of organisms. We also discovered potential key metabolites produced by ELF that are yet to be reported. This study shows the application of metabolomics in rapidly identifying bioactive metabolites that are of significance in the discovery of new drugs.

**Abstract:**

Three species of the lichen *Usnea* (*U. baileyi* (Stirt.) Zahlbr., *U. bismolliuscula* Zahlbr. and *U. pectinata* Stirt.) and nine associated endolichenic fungi (ELF) were evaluated using a metabolomics approach. All investigated lichen crude extracts afforded antibacterial activity against *Staphylococcus aureus* (minimum inhibitory concentration (MIC): 0.0625 mg/mL), but none was observed against *Escherichia coli*, while the ELF extract *Xylaria venustula* was found to be the most active against *S. aureus* (MIC: 2.5 mg/mL) and *E. coli* (MIC: 5 mg/mL). *X. venustula* was fractionated and tested for to determine its antibacterial activity. Fractions XvFr1 to 5 displayed bioactivities against both test bacteria. Selected crude extracts and fractions were subjected to metabolomics analyses using high-resolution LC–MS. Multivariate analyses showed the presence of five secondary metabolites unique to bioactive fractions XvFr1 to 3, which were identified as responsible for the antibacterial activity of *X. venustula.* The *p*-values of these metabolites were at the margin of significance level, with methyl xylariate C (P_60) being the most significant. However, their high variable importance of projection (VIP) scores (>5) suggest these metabolites are potential diagnostic metabolites for *X. venustula* for “dual” bioactivity against *S. aureus* and *E. coli.* The statistical models also showed the distinctiveness of metabolites produced by lichens and ELF, thus supporting our hypotheses of ELF functionality similar to plant endophytes.

## 1. Introduction

As symbiotic organisms, lichens are known as unique sources of bioactive secondary metabolites called lichen substances [1]. The mycobiont, which is the fungal component of the lichen, is reported to produce approximately 1000 chemically diverse lichen substances [2]. Most of these metabolites are unique to lichens, with only a small portion occurring in fungi and higher plants [3]. These substances were found to exhibit significant biological activities including antibacterial [4], antifungal [5], anti-inflammatory [6], antioxidant [7], antiviral [8] and cytotoxic [9] activities. Nevertheless, their slow growth in nature limits the widespread application of these substances, particularly for pharmaceutical uses [10]. In addition, overcollection of lichens impacts their conservation. Hence, other fast-growing and easily cultured organisms, in contrast to slow-growing lichens, are prioritized. Interestingly, a group of asymptomatic fungi that resemble plant endophytes was discovered thriving inside healthy lichen thalli [11,12]. This group of fungi, known as endolichenic fungi (ELF), was first described in 1990 [13]. In our other study, we reported very diverse species of ELF from *Usnea* which differed significantly between geographic origins—e.g., Philippines vs. Malaysia [14,15]. Since then, over 35 species of ELF have been isolated and characterized [16]. Furthermore, ELF are also known to possess secondary metabolites that are hypothesized to be distinct from those produced by lichens [17]. Few studies, however, have been conducted to specifically view such distinction. Since the first report on the metabolites produced by ELF in 2007 [18], a tremendous increase in the number of compounds has been described. To date, 351 secondary metabolites produced by ELF have been characterized and identified, 196 of which were novel [16]. These metabolites consisted of a diverse array of structural types that included alkaloids, quinones, oxygenated heterocycles, aromatic compounds, terpenes, peptides, steroids, and allylic compounds, which were also known to exhibit various biological activities [19,20,21,22,23]. ELF have therefore offered another avenue for drug discovery.

The search for new bioactive natural products for drug discovery has always been a challenge. Metabolomics, a comprehensive profiling tool used to study and quantify small molecules present in a biological system [24], is gaining popularity in speeding up the discovery of active and novel drugs. It is considered as the most recent member of the “omics” technology, which involves advanced instrumentation, analytical, and statistical methodologies and well-established metabolic pathways and large metabolite databases [25]. An integral component of metabolomics, called dereplication, is the recognition and elimination of already known substances in the early stages of the screening process in a drug discovery scheme [26]. Through dereplication, the discovery of bioactive and novel natural products is accelerated [27]. 

In this study, a metabolomics approach was adopted to identify potentially novel and active secondary metabolites from the three species of *Usnea* and their associated ELF. Using the generated LC–MS data, statistical multivariate data analyses, i.e., principal component analysis (PCA), partial least squares discriminant analysis (PLS-DA) and orthogonal projection to latent structures discriminant analysis (OPLS-DA), were carried out to capture the significant metabolites produced uniquely by the host lichen *Usnea* and its associated ELF with antibacterial activities against *Staphylococcus aureus*. The obtained data of this study were analyzed and integrated to prioritize the specific lichen and ELF producing interesting compounds for future isolation work. Finally, this study also aimed to compare the chemical profiles and antibacterial properties of lichens and ELF crude extracts, which may provide an interesting insight on the possible role of ELF in their lichen hosts.

## 2. Materials and Methods

### 2.1. The Test Organisms: The Lichen Usnea and the Isolated Endolichenic Fungi (ELF)

Three lichens, *U. baileyi* (Stirt.) Zahlbr., *U. bismollliuscula* Zahlbr., and *U. pectinata* Stirt., were collected from barks of pine trees (*Pinus merkusii*) growing dominantly in Bukit Larut in Perak, Malaysia (4°51′44′′ N, 100°47′36′′ E) and Sagada in Mountain Province, Philippines (16°5′30′′ N, 120°50′48′′ E). This pine tree species is native to the Philippines but was introduced in Peninsular Malaysia in the 1950s for reforestation [28]. These trees, approximately >30 years old, were found in nonplantation areas. Permission to collect lichens was obtained from nearby land owners. Collection of lichens on the tree trunk was carried out one meter from the ground and only those lichens with lengths of >1.5 inches were collected. Extra care was taken to ensure that no trees were wounded or injured during the collection. These lichen specimens were placed in sterile plastic bags and properly labeled with location, elevation, and global positioning system (GPS) data. These lichens were processed within 24 to 48 h. From these lichens, ELF were isolated following the protocol of Samanthi et al. [21] with modifications of the ethanol concentration and an additional step of plating the rinsed water onto Potato Dextrose Agar (PDA; Merck, Darmstadt, Germany) to validate the efficacy of the surface sterilization. Briefly, the lichen thalli were surface-sterilized by successive dipping on various ethanol concentrations (70%, 80%, 85%, and 90% v/v) for 1 min, and then in 10% sodium hypochlorite (NaOCl) for 30 s and 95% ethanol for 30 s. The surface-sterilized lichen thalli were cut into small fragments and plated onto 2% malt yeast extract agar (MYE, Lab M Limited, Heywood, UK). The ELF isolates were then identified by sequence analysis of the ITS region of the rRNA and deposited on GenBank under specific accession numbers. Specific details on the isolation, identification, and diversity assessment of the lichen hosts and the associated ELF have been separately reported in our other studies [14,15].

### 2.2. Extraction of Secondary Metabolites Produced by Lichen Usnea and ELF

Six lichen specimens, three representing each study site, and nine ELF isolates, were extracted for their secondary metabolites (Table 1). These ELF isolates were selected based on their abundance, unique morphologies, and relatively short incubation periods (i.e., <7 days). The extraction of lichen acids was carried out following the protocol of Santiago et al. [29]. Briefly, one gram of each lichen sample was ground until powdery then macerated with 10 mL of acetone overnight. The crude extract was filtered using a sterile filter paper (Whatman no. 1, Tisch Scientific, North Bend, OH, USA) and the supernatant was collected in a preweighed vial. Each vial was carefully covered with a sterile cheese cloth until the acetone evaporated completely to yield the crude extract. For the extraction of the ELF secondary metabolites, a solid-state fermentation was carried out. Each ELF isolate was initially grown on PDA for 15 days under light at 26 ± 2 °C for 12 h. After incubation, 10 fungal agar blocks (~15 mm in diameter) were inoculated in 500 mL Erlenmeyer flasks containing 200 mL of Glucose Malt Yeast Extract (GMY; Lab M Limited, Lancashire, UK) (9 × 200 mL) broth and incubated at 26 ± 2 °C on a rotary shaker (135 rpm) for 15 days at room temperature (26 ± 2 °C) to obtain the seed culture. Scale-up fermentation was carried out for all nine ELF strains in five 1 L Erlenmeyer flasks (five replicates), each containing 160 grams of rice and 240 mL of distilled water [30]. The rice media were then autoclaved, and each flask was inoculated with 25 ml of the seed culture and incubated at 26 ± 2 °C for 50 days. 

### 2.3. Antibacterial Activities of the Lichen and ELF Crude Extracts

Paper disk diffusion and agar well assays (File S1) were used to evaluate the antibacterial properties of lichen and ELF crude extracts, respectively, following Clinical and Laboratory Standards Institute (CLSI) recommendations [31]. The test organisms used were *Escherichia coli* ATCC 25922 and *Staphylococcus aureus* ATCC 25923. The antibacterial activities of the crude extracts presented in this paper have also been reported in our recent works [14,15]. However, the inclusion of these data in the current metabolomic analyses is necessary to compare the bioactivities of the crude extracts with those of their respective fractions. In addition, the minimum inhibitory concentrations (MICs) and minimum bactericidal concentrations (MBCs) of all crude extracts were also determined (File S1). The MIC and MBC values presented in this study were reported in our earlier works [14,15]. These data are again presented here to illustrate the selection criteria for the succeeding fractionation works.

### 2.4. Selection of Lichen and ELF Crude Extracts for Fractionation Work

Two criteria for selecting lichen and ELF crude extracts were considered in this study: (1) high metabolic diversity and (2) moderate to strong antibacterial activities. For the assessment of the metabolic diversity, analysis of the mass spectra of the crude extracts was carried out using MetaboAnalyst© 4.0 [32]. Key features of this process, which are incorporated in the software, include data upload and cleaning, processing, normalization, statistical analyses, and annotation. Heatmaps, together with hierarchical clustering techniques, were generated using the software’s default settings. For the antibacterial activities, extracts with zones of inhibition (ZOIs) of ≥10 mm and low MIC and MBC values (≤10 mg/mL) were considered. Selection of crude extracts to be fractionated resulted in a small sample size of this study. As such, possible biases during analyses may be present. Nevertheless, the data presented in this study may provide preliminary information regarding the metabolomics analyses of lichen and ELF crude extracts. 

### 2.5. Fractionation and Initial Characterization of Selected Lichen and ELF Crude Extracts 

Lichen and ELF crude extracts that exhibited moderate to strong antibacterial activities (≥10 mm ZOI) were selected for fractionation using silica gel column chromatography. Thin layer chromatography (TLC) was first carried out with aluminum precoated silica gel 60 F_254_ plates (Merck, Darmstadt, Germany). Spots were then visualized under UV light (λ = 254 nm). Following TLC, silica gel chromatography (silica gel 60, Merck, Darmstadt, Germany) was performed. An isocratic system of toluene/acetic acid (85:15) was used as the mobile phase for lichen crude extracts. The ELF crude extracts were fractionated using a gradient of dichloromethane and methanol (DCM/MeOH, 90:10, 80:20, 70:30, 60:40 and 50:50), with solvent volume ranging between 200 and 1000 mL until the collected fraction was rendered colorless. All solvents used were of analytical grade. Following fractionation, the lichen and ELF crude extracts and fractions, including usnic acid and the solvents acetone and ethyl acetate, were subjected to high-resolution LC–MS (LC-HRMS) analysis. LC–MS was carried out using an Agilent 1290 Infinity LC system coupled to an Agilent 6520 Accurate-Mass-Q-TOF mass spectrometer with dual ESI source instrumentation (California, USA) equipped with Agilent Zorbax Eclipse XDB-C18 (Santa Clara, CA, USA). A 2.1 × 150 mm column was used with the following parameters: column temperature at 25 °C, 0.5 mL/min flow rate, and 10 µL injection volume. A concentration of 1 mg/mL of each sample was used. The mobile phase consisted of 0.1% formic acid in water and 0.1% formic in acetonitrile. High-resolution mass spectrometry (HRMS) was carried out in both positive and negative ionization modes. The analysis had a run time of 25 min and a postrun time of 5 min. Data generated from LC–MS were used in metabolomics analyses. A single technical replicate was used in this study. Furthermore, the antibacterial activities of the lichen and ELF fractions were also used (see Section 2.3).

### 2.6. Metabolomics Profiling Studies

LC–MS data were used for metabolomic profiling studies following the protocol of Macintyre et al. [33]. Raw data were first converted to mzML file format using the software ProteoWizard [34]. Data processing was carried out using the software MZMine 2 [35]. Key features of this process included detection (mass detector: centroid; noise level: 1000; minimum time span: 0.2 min; minimum height: 1 × 10^4^; *m*/*z* tolerance: 0.001 *m*/*z* or 5 ppm,), deconvolution (chromatographic threshold: 5%), deisotoping (*m*/*z* tolerance: 0.001 *m*/*z* or 5 ppm, retention time (RT) tolerance: 0.1 absolute (min), maximum charge: 2, representative isotope: most intense), filtering (*m*/*z* range: 100–1999, RT: 0–45 min), alignment (*m*/*z* tolerance: 0.001 *m*/*z* or 5 ppm, *m*/*z* weight: 20, RT tolerance: 5 relative %) and gap filling (*m*/*z* tolerance: 0.001 *m*/*z* or 5 ppm, intensity tolerance: 1%). Complete software settings were as described by Macintyre et al. [33]. A data clean-up was also carried out by creating an Excel macro that enabled positive and negative ionization data (exported as CSV file) to be processed together, minimizing the risk of missing poorly ionizing compounds only detected in one mode. The Excel macro was used to dereplicate the samples, matching each *m*/*z* found in the samples with those in the Dictionary of Natural Products (DNP, version 2017) database to provide complete information of all the putative identified metabolites, as well as those that were unidentified. For the dereplication, a feature ID number, ionization mode, *m*/*z*, retention time, possible molecular formula, peak intensity, related compounds, and source of the specific metabolites (if available) were generated. 

### 2.7. Multivariate Analyses

Multivariate analyses (MVAs) that included both Principal Component Analysis (PCA), Partial Least Squares-Discriminant Analysis (PLS-DA) and Orthogonal Projection to Latent Structures Discriminant Analysis (OPLS-DA) for the prediction of antibacterial metabolites were carried out using the software SIMCA ver. 15.0.2 (Umetrics, Umeå, Sweden) and MetaboAnalyst© 4.0 [32]. PCA was used to observe an overview of variance between the lichen and ELF crude extracts (predictor variables) and secondary metabolites (responses) generated from LC–MS data and to detect outliers that could influence the model. PLS-DA and OPLS-DA, on the other hand, were used to investigate the significant differences among metabolites produced by two different taxonomic origins (i.e., lichen and ELF) and their respective bioactivities (i.e., active and inactive samples against *S. aureus*), respectively. OPLS-DA was also used to identify the chemically distinct samples that may yield novel and bioactive secondary metabolites. A pareto scaling was applied to all MVA to reduce the influence of intense peaks while emphasizing weaker peaks that may have more biological relevance [36]. The Q^2^ and R^2^ values were reported as a qualitative measure of consistency between the predicted and original data. These values explained the goodness of prediction of the statistical models, representing the total explained variance and the predictive power of the models. The CV-ANOVA was also employed for further statistical validity of the models. The variable importance of projection or VIP scores were determined using the SIMCA software. This further validates the “ultimate” discriminating features defined on the PLS-DA plot, identifying the metabolites that greatly contribute to the variation of the groups. The greater the VIP score, the higher the discriminatory power of the metabolite. VIP scores >1 show the metabolites carrying highly discriminating information between classes [37]. 

## 3. Results

### 3.1. Bioactivities of Lichen and ELF Erude Extracts

Crude extracts of the lichen *Usnea* and their ELF were evaluated for antibacterial properties. These antibacterial data were separately reported and discussed in detail in our other studies [14,15]. In general, lichen crude extracts only showed inhibition against *S. aureus*, with *U. bismolliuscula* collected from the Philippines (PH) showing the highest ZOI followed by *U. baileyi* (PH). All lichen crude extracts gave a similar MIC value of 0.0625 mg/mL (Table 2). Meanwhile, six of the nine ELF crude extracts were active against *S. aureus* and *E. coli*, with *Annulohypoxylon albidiscum* and *Xylaria venustula* exhibiting the strongest activities against *S. aureus*, albeit having a high MIC value of 2.5 mg/mL. Interestingly, *X. venustula* also exhibited activity against *E. coli*, with a MIC value of 5 mg/mL (Table 2). 

### 3.2. Metabolomic Profiles of Lichen and ELF Crude Extracts

**Figure 1:**
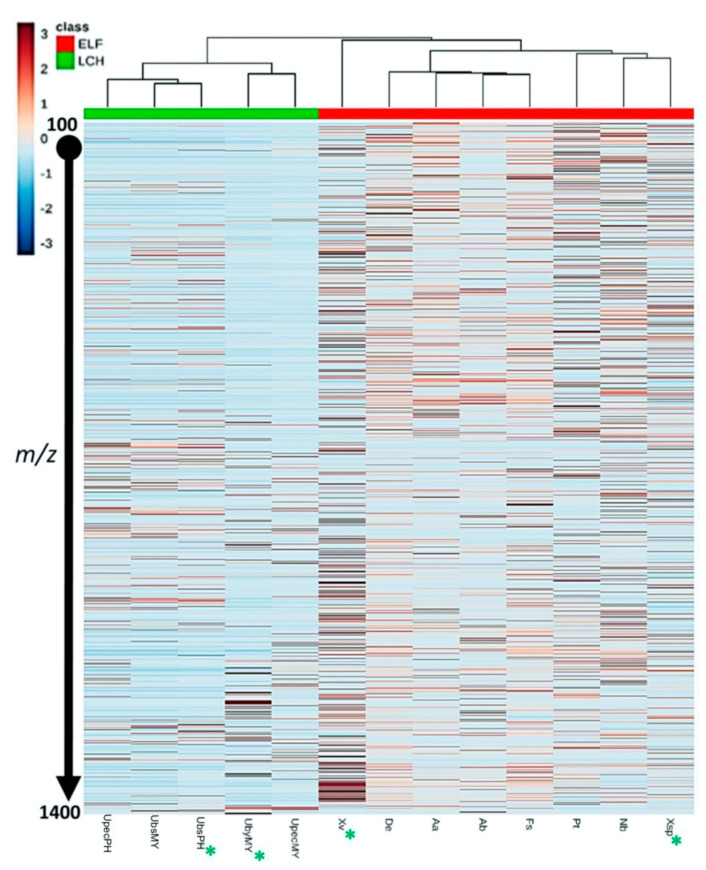
Heatmap and dendrogram of crude lichen and ELF extracts. The presence of more red bars indicates higher diversity in their chemical profile. Those marked with an asterisk (*) were chosen for further fractionation work (ELF: endolichenic fungi, LCH: lichen).

The crude extract of *U. baileyi* (MY, UbyMY) yielded a higher metabolite density and diversity, with molecular weights ranging from 400 to 1400 Dalton (Da) as indicated by the heatmap (Figure 1). However, no further metabolite profiling was carried out on *U. baileyi* collected from the Philippines (PH) due to very low yield of extract that was less than a milligram. *U. pectinata* samples (UpecMY and UpecPH), on the other hand, also revealed comparable densities and diversity of metabolites in the same molecular weight range. The molecular weights of the metabolites present in UpecPH ranged between 200 and 1400 Da (Figure 1). However, the heatmap, along with the PCA scores plot (Figure S1), revealed that UpecPH and UpecMY gave different chemical profiles as they were situated farther from each other on the dendrogram (Figure 1) and their cluster sphere (Figure S1). Furthermore, crude extracts of *U. bismolliuscula* collected from MY (UbsMY) and PH (UbsPH) gave very similar and almost identical chemical profiles, as indicated by both heatmap-dendrogram and PCA scores plot, where both samples clustered together. Molecular weights of the metabolites detected in UbsMY and UbsPH ranged between 250 and 1400 Da (Figure 1). The MVA model gave an R^2^ value of 0.99, a Q^2^ value of 0.72, and accuracy of 1 using Pareto scaling at two components.

In general, ELF crude extracts indicated a higher diversity in their metabolomic profiles when compared to their lichen hosts. Among the ELF crude extracts, *Xylaria venustula* (PH, Xv) afforded the most diverse metabolomic profile as shown on the heatmap-dendrogram (Figure 1). This was followed by *Pseudopestalotiopsis theae* (MY, Pt), *Nemania bipapillata* (PH, Nb) and *Xylaria* sp. (MY, Xsp). Molecular weights of the metabolites found in these four ELF isolates ranged between 100 and 1400 Da. Interestingly, both Xsp and Xv were separated from the main cluster of other ELF samples as shown by the scores plot (Figure S1), indicating their unique chemical profiles. 

### 3.3. Bioactivities of Lichen and ELF Fractions

**Figure 2:**
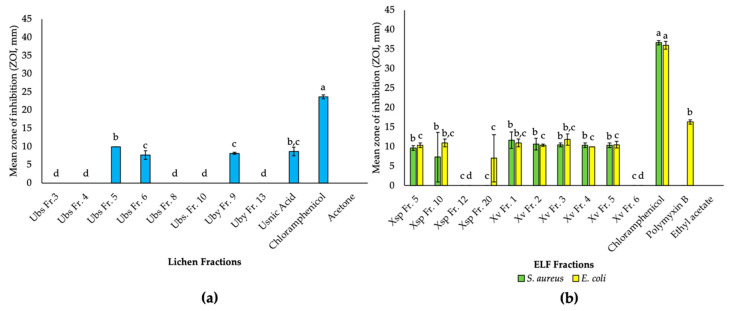
Biological activities of lichen and ELF fractions. (**a**) Antibacterial activities of *U. bismolliuscula* (PH, UbsPH) and *U. baileyi* (MY, UbyMY) fractions against *S. aureus* ATCC 25923. (**b**) Antibacterial activities of *Xylaria* sp. (MY, Xsp) and *X. venustula* (PH, Xv) fractions against *S. aureus* ATCC 25923 and *E. coli* ATCC 25922. Standard deviation values are indicated by the error bars (*p* < 0.05). Letters above error bars indicate the statistical significance within groups using one-way ANOVA and Tukey HSD. a, b, c, d different letters indicated on the columns denote statistical significance between respective fractions.

In this study, the crude extracts of *U. bismolliuscula* (UbsMY and UbsPH), *U. baileyi* (UbyMY) and *U. pectinata* (PH, UpecPH) offered relatively diverse chemical profiles. In terms of their antibacterial activities, UbsPH exhibited the strongest activity against *S. aureus* (16 mm ZOI) among all the lichen crude extracts, followed by UbyMY (12 mm ZOI). Therefore, UbsPH and UbyMY were chosen for further fractionation work affording six and four fractions, respectively. From the UbsPH fractions, UbsFr5 was found to be active (10 mm ZOI), followed by UbsFr6 (7.7 mm ZOI), while the rest of the other tested fractions did not manifest any inhibition (Figure 2a). Interestingly, UbsFr5 exhibited higher inhibition than the reference usnic acid. From UbyMY, only UbyFr9 was found to be active against *S. aureus* (8.2 mm ZOI). 

In this study, Xv and Xsp were the most abundant isolated ELF. Xv also produced the highest extract yield of 9.8 g (1.22%), followed by Xsp with 3.9 g (0.48%) (Table 1). For their bioactivities, Xv gave MIC and MBC values of 2.5 and 5 mg/ml against *S. aureus* and *E. coli*, respectively (Table 2). Xsp, on the other hand, only gave an MIC of 10 mg/mL against both test bacteria. There were also other ELF isolates, such as *Xylariaceae* sp. (Xcsp) and *Annulohypoxylon albidiscum* (Aa), that exhibited even stronger antibacterial activities than Xv and Xsp. However, these isolates only exhibited activities against *S. aureus*, and hence, were not prioritized in this study. In addition, Xcsp was discontinued due to its poor growth reproducibility in the flask cultures. The chemical profile of Aa also afforded less diversity of compounds. Due to chemical diversity, dual activity against both test organisms, as well as good yield of extracts, further fractionation was achieved with Xsp and Xv yielding seven and six fractions, respectively. From Xsp, only the fractions XspFr1 and XspFr10 were found to be active against both *S. aureus* and *E. coli* (Figure 2b). Similarly, fractions XvFr1 to 5 from Xv also showed significant activities against both test strains (Figure 2b). Low yielding fractions were not feasible for any of the assays used in this study.

### 3.4. Chemical Profile Differences between Host Lichens and Associated ELF

**Figure 3:**
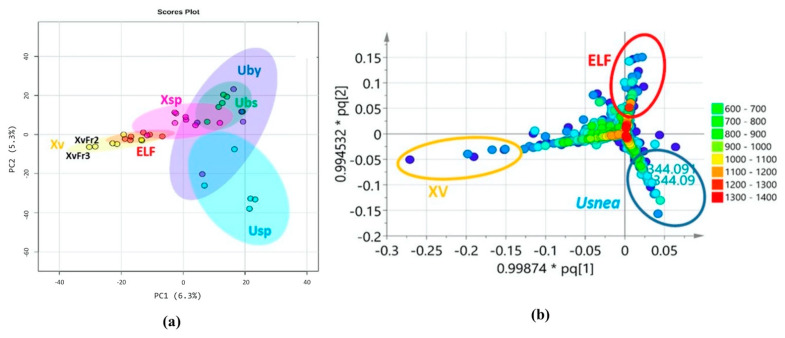
Multivariate analyses of the LC–MS data of 36 lichen and ELF crude extracts (including the reference (+)-usnic acid) and selected fractions. (**a**) principal component analysis (PCA) scores plot revealed distinct Xv fractions away from the overlapping clusters. (**b**) Encircled features on partial least squares discriminant analysis (PLS-DA) loading plot generated from SIMCA revealed metabolites (in molecular weight) responsible for the separation of the respective taxonomical groups. (ELF: endolichenic fungi, UBS: *U. bismolliuscula,* UBY: *U. baileyi,* USP: *U. pectinata,* XSP: *Xylaria* sp., XV: *X. venustula*). The pq vectors indicate 99.87% and 99.45% influence of individual X- and Y-variables, respectively, to the model suggesting very good prediction of underlying patterns in the data.

Mass spectral data of 36 lichen and ELF crude extracts and fractions were subjected to MVA. Usnic acid was used as a reference standard to validate the positioning of the *Usnea* samples in the various MVA plots. PCA of the lichen and ELF crude extracts and fractions gave a good separation between the host lichen and their associated fungi. However, in the PCA plots, all ELF samples clustered together except for the outlying samples originating from *X. venustula* (Xv). The score scatter plot (Figure 3a) revealed two distinct fractions, XvFr2 and XvFr3. A heatmap (Figure S2) displays the comparative distribution of the metabolites ranked by t-tests in the respective classes and indicated the presence of a set of collective metabolites for the antibacterial extract Xv along with its bioactive fractions XvFr2, XvFr3 and XvFr4 (in yellow box, Figure S2). It was also observed that the *Xylaria* samples (Xsp and Xv) afforded a unique set of metabolites, which did not align with those of the other ELF samples as further shown on the heatmap (in blue box, Figure S2). The occurrence of usnic acid in *Usnea* extracts (UpecMY, UpecPH, UbsMY, and UbsPH) representing the lichens *U. pectinata* and *U. bismolliuscula* was also be revealed on the heatmap (Figure S2). However, usnic acid was not detected in *U. baileyi* (UbyMY). 

A similar result was amplified by the PLS-DA loading plot generated from SIMCA (Figure 3b), showing clearly the discriminating metabolites that differentiated the *Usnea* host lichens from its associated ELF, as well as the separation of the *Xylaria* samples from the rest of the ELF strains investigated in this study. The occurrence of usnic acid with the molecular weight of 344.09 Da specified the clustering of the *Usnea* samples. The PLS-DA loading plot (Figure 3b) disclosed unique discriminating metabolites with molecular weights of 100 to 300 Da representing the *Xylaria* groups, while the rest of the ELF samples covered molecular weights of 200 to 400 Da. However, their *Usnea* hosts yielded metabolites with higher molecular weights of 300 to 500 Da. The metabolites that separated the fractions of Xv were the same as those that discriminated the distinct position of XvFr2 and XvFr3 on the PCA scores plot. The PLS-DA loading plot (Figure 3b) also displayed the molecular ion masses of the secondary metabolites responsible for discriminating XvFr2 and XvFr3. 

**Figure 4:**
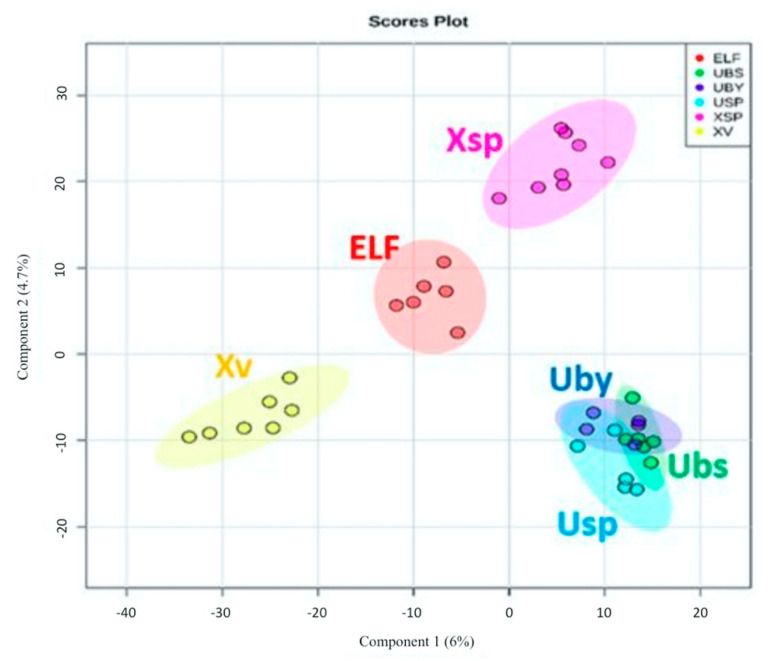
PLS-DA scores plot of lichen and ELF fractions grouped with their respective crude extracts and according to their taxonomical origin. (ELF: endolichenic fungi, UBS: *U. bismolliuscula*, UBY: *U. baileyi*, USP: *U. pectinata*, XSP: *Xylaria* sp., XV: *X. venustula*).

In a PLS-DA scores plot (Figure 4), lichen and ELF crude extracts and fractions were grouped according to their taxonomical origins. The model achieved an R^2^ value of 1, a Q^2^ value of 0.56, and accuracy of 0.81 using Pareto scaling at five components. The PLS-DA score scatter plot afforded the separation of four distinct groups representing the extracts of the lichen hosts *U. baileyi* (Uby), *U. bismolliuscula* (Ubs), and *U. pectinata* (Usp) on one group, while their associated ELF along with their respective fractions were divided into three clusters (ELF, Xsp and Xv) (Figure 4). *Xylaria* sp. (Xsp) and *X. venustula* (Xv) extracts together with their fractions were separated from the ELF cluster. SIMCA found a variation of 12.5% for the separation between groups while the intermodal variation was only 3.15%, indicating a relatively good similarity in chemical profiles within respective clusters. A heatmap and dendrogram of lichen and ELF extracts and fractions (Figure S3a) based on the top 25 metabolites ranked according to their Variable Importance of Projection (VIP) scores were generated to validate the “ultimate” discriminating features from the PLS-DA loading plot (Figure 3b). These discriminating features demarcated the groupings of the samples into clusters shown on PLS-DA scores plot (Figure 4). The dominance of the Xv metabolites was illustrated on the VIP score heatmap for the top 25 metabolites (Figure S3a), which again established the richness and unique chemical diversity that *X. venustula* offered. The top 15 metabolites were listed on a VIP score scatter plot indicating their relative abundance in their respective classes (Figure S3b).

**Figure 5:**
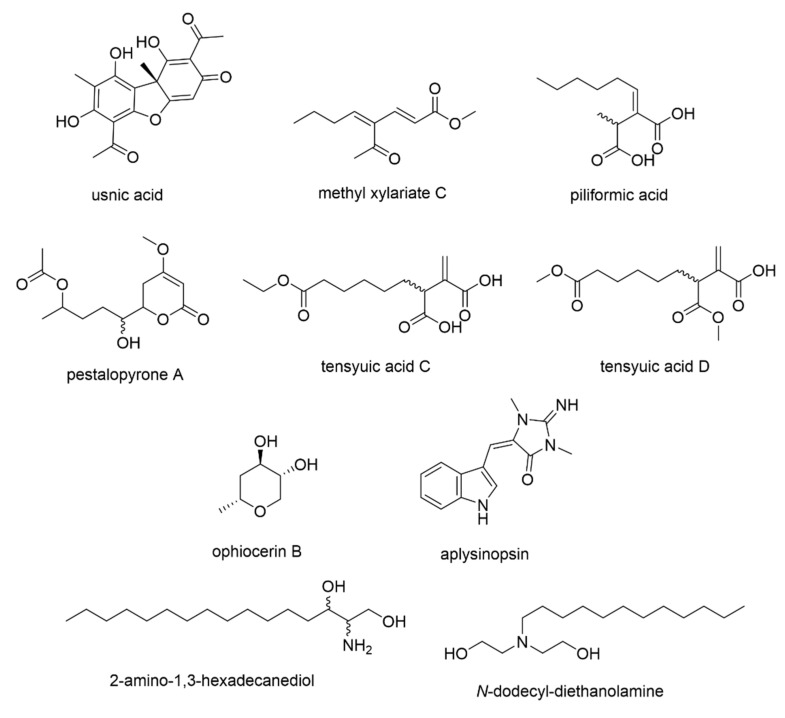
Structures of putatively dereplicated metabolites with usnic acid used as reference standard for lichen extracts in this study.

The discriminating features for the bioactive Xv extract and fractions were among those with VIP scores >5 that included *m*/*z* (VIP scores) *m*/*z* [M+H]^+^ 197.1173 (9.83), [M+H]^+^ 255.1233 (6.65), [M−H]^−^ 195.1031 (6.54), and [M+H]^+^ 273.1336 (5.07), which were all primarily detected from the PLS-DA loading plot (Figure 3b) and dereplicated from the DNP database (Figure 5, Table 3). Ion peaks at *m*/*z* [M+H]^+^ 197.117 and 215.128 were identified to be the Xylaria metabolites and methyl xylariate C and piliformic acid, respectively. The ion peaks at *m*/*z* [M−H]^−^ 131.071 and [M+H]^+^ 273.134 were dereplicated as the fungal natural products ophiocerin B and tensyuic acid C/D or pestalopyrone A, respectively. The ion peaks at *m*/*z* [M+H]^+^ 255.123 and 274.274 were identified from the database as the sponge metabolites aplysinopsin and 2-amino-1,3-hexadecanediol or the marine-derived bacterial metabolite N-dodecyl-diethanolamine, respectively. 

### 3.5. Predicting the Bioactive Metabolites

**Figure 6:**
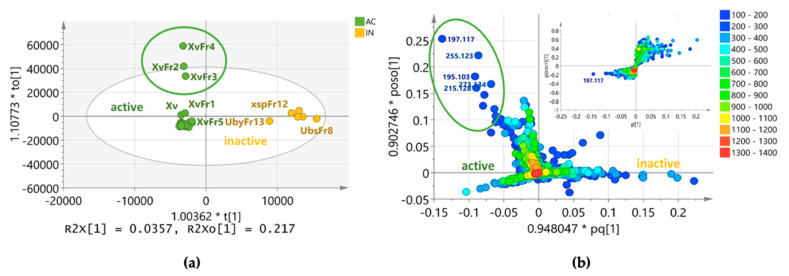
Multivariate analyses of 36 lichen and ELF crude extracts and selected fractions to predict the antibacterial metabolites in the *Xylaria* samples (Xsp and Xv). (**a**) Orthogonal projection to latent structures discriminant analysis (OPLS-DA) score scatter plot of samples categorized according to their bioactivities. (**b**) OPLS-DA loadings and S-plots with encircled features represent the bioactive metabolites of the most active samples. Active metabolites are on the left quadrants while inactive features are on the right quadrants. The pq and poso vectors indicate 94.80% and 90.27% influence of individual X- and Y-variables, respectively, to the model suggesting good prediction of underlying patterns in the data.

LC–MS data of the 37 samples were subjected to OPLS-DA (Figure 6) to predict the secondary metabolites responsible for their bioactivities. The samples were grouped into two classes: active vs. inactive (Figure 6a). The OPLS-DA score scatter plot gave an R^2^ value of 0.976 and a low Q^2^ value of 0.0558. The fractions XvFr3 and XvFr4 were revealed as the outliers while XvFr3 just clustered towards the latter (Figure 6a). The model gave a good fit but very poor predictive power. This was due to the higher intermodal variation (R^2^Xo) of 21.7% while the variation between two groups (R^2^X) was only 3.57%, indicating distinct differences in chemical profiles between the samples within the respective classes (Figure 6a). In parallel, the OPLS-DA loading plot (Figure 6b) displayed the secondary metabolites (P_60, N_592, P_1587, P_1117 and P_1588) assumed to be responsible for the bioactivity, as well as for discriminating the outliers (Table 3). These metabolites were among the discriminating features earlier defined in the previous section for the uniqueness of the Xv extract and fractions. The additional ion peak at *m*/*z* 195.102 eluting at 11.26 min is the negative ion [M−H]^−^ of P_60. 

From the S-plot, the end features pinpointed the metabolites P_60, N_592, P_1587, P_1117, and P_1588 responsible for the antibacterial activity of *X. venustula,* which achieved *P*-values between 0.05 and 0.2, with at least 80% confidence against the null hypothesis (Table 3). P_60 with a mass ion peak at *m*/*z* [M+H]^+^ 197.117 and a molecular weight of 196.11, was dereplicated as 4-acetyl-2,4-octanedienoic acid (methyl xylariate C) afforded a significant *P*-value of 0.05. The exclusive occurrence of P_60, N_592, P_1587, P_1117, and P_1588 in the bioactive extract and fractions were further validated with box-and-whisker plots (Figure S4a) and their VIP scores were all >5 (Figure S4b).

## 4. Discussion

In this study, the chemical profiles and antibacterial activities of different species of *Usnea* and their associated ELF strains were evaluated using metabolomics tools. Three species of *Usnea* collected from Malaysia and the Philippines—*U. baileyi, U. bismolliuscula*, and *U. pectinata—*were evaluated on their antibacterial activities. Consistent with previous studies [29,38,39], results revealed that all lichen crude extracts were active against Gram-positive bacteria but inactive against Gram-negative bacteria (as reported in Santiago et al. [14,15]), albeit we only tested one representative bacterial species per category. Usnic acid, the major secondary metabolite found in the lichen *Usnea,* is known as the active substance responsible for uncoupling oxidative phosphorylation in bacteria, leading to cell death [40]. Other bioactive lichen metabolites such as norstictic and salazinic acids have also been reported in the genus *Usnea* [29]. In this study, however, only usnic acid was used as the reference lichen substance. A slight difference in the bioactivities in terms of the size of the ZOI was observed in this study between extracts of similar *Usnea* species collected from different sites. This could be due to quantitative differences in secondary metabolites produced by the respective lichens, which have consistently been described among lichen populations [41]. ELF crude extracts were found to be active against both the Gram-positive *S. aureus* and Gram-negative *E. coli* [14,15]. Although limited test organisms were evaluated in this study as we only wanted to initially explore their potential bioactivities, ELF were found as good sources of bioactive compounds and may offer medicinal and industrial advantages over their lichen hosts. Similar observations were reported by Padhi and Tayung [42]. 

In relation to the crude extracts, fractions obtained from lichen hosts and ELF that exhibited antibacterial activities were evaluated and compared. As expected, lichen fractions were only active against *S. aureus,* while few ELF fractions were active against both test bacteria. There were, however, fractions that did not exhibit any activities against the test bacteria, despite the strong antibacterial activity of its crude extract. This could be due to the absence of other features in the crude extract found earlier that behaved synergistically and, as a result of fractionation, the metabolites were separated from each other, hence resulting in the loss of bioactivity [43]. 

In this study, PCA, an unsupervised MVA method, was performed using LC-HRMS data. The score scatter plot gave an overview of the samples’ chemical profiles, while determining the relationship between groups [36]. In this study, the generated PCA showed the groupings of samples based on their secondary metabolite profiles. R^2^ at *ca.* 0.99, Q^2^ > 0.72, and an accuracy of 1 suggested good fit and predictive power of the model. The generated score scatter plot revealed very good separation between the lichen hosts and their associated ELF. The *Xylaria* samples, Xv and Xsp, were distinctly situated farthest from the main ELF cluster, which suggests the uniqueness of their chemical profiles. Such an observation supports the hypothesis that ELF produce metabolites distinct from their lichen hosts [17].

PCA and PLS-DA of the fractions, along with their respective extracts, were also performed. In this study, unique secondary metabolites were identified from the ELF *X. venustula*. All of these metabolites, to the best of our knowledge, are yet to be reported for ELF. The discriminating features of the Xv fractions were caused mainly by six secondary metabolites (P_60, P_844, P_1117, P_1587, P_1588, and N_215). The metabolites P_60 and P_1117 represented chemical uniqueness for the distinct Xv fractions, thus prompting their separation from the rest of the ELF extracts and fractions. P_60 and P_1117 were putatively identified as methyl xylariate C (or 4-acetyl-2,4-octadienoic acid) and piliformic acid, respectively. These were first isolated from an endophytic fungus *Xylaria* and reported to exhibit antimicrobial and cytotoxic activities [44,45]. As such, identifying these compounds from an endolichenic fungus *Xylaria* is feasible. In addition, it was previously reported that ELF produce secondary metabolites with carbon skeletons similar to those of the bioactive compounds produced by endophytes and are hypothesized to perform similar ecological roles for the host organism [17]. As such, similar gene clusters are expressed, resulting in the production of comparable metabolites by both endophytic and endolichenic fungi belonging to the same family—e.g., Xylariaceae. Furthermore, the similarity in their carbon skeletons despite being produced by different host organisms may also result in similar biological properties as observed in previous studies [45].

The metabolite P_1588 was dereplicated as pestalopyrone A and tensyuic acid C or D. The antifungal agent pestalopyrone A was first described from the endophytic fungus *Pestalotiopsis microspora* [46]. On the other hand, tensyuic acids (C and D) were previously isolated from the terrestrial fungus *Aspergillus niger* and were reported to have moderate antibacterial activities against *Bacillus subtilis* [47]. The tensyuic acid derivatives are acyclic congeners of pestalopyrone A. Interestingly, the structure of piliformic acid is also very closely related to tensyuic acid where the alkyl chain is attached on the exomethylene unit.

Aplysinopsin (P_1587) is a tryptophan derivative that was first isolated from the marine sponge genus *Thorecta* [48], and has been isolated from other marine invertebrates [49]. This compound has been reported for its broad spectrum of bioactivities such as antimicrobial, antiparasitic, and cytotoxic activities [49]. Additionally, congeners of 2-amino-1,3-hexadecanediol (P_844) have been isolated from marine-derived bacteria and other marine invertebrates [50]. Alternatively, P_844 was also dereplicated as *N-*dodecyl-diethanolamine (DDE) and has been described as displaying a broad spectrum of activity towards *Escherichia coli,* which may have been influenced by the formation of micelles [51]. These latter compounds were never reported in the literature as lichen or fungal metabolites from terrestrial sources, implying that these discriminating ion peaks could also belong to metabolites structurally different to the dereplicated compounds and could instead be plausibly rendered as new natural products.

In this study, it can be observed that most identified metabolites were previously reported from other organisms such as plants, soil fungi (pathogens and endophytes), and marine organisms. It was has been postulated that ELF is a precursor to major ecological transitions such as pathogenicity, symbiosis, and endophytism [11]. As such, isolation of metabolites previously reported from endophytic or pathogenic fungi may also be expected for ELF. Their metabolic pathways are alike; hence, production of similar secondary metabolites is feasible.

OPLS-DA was employed to predict the secondary metabolites responsible for the antibacterial activities of all samples by grouping them between active and inactive. The model’s R^2^ was 0.976, indicating a good fit. The results were interpreted and it was found that 24.2% of the x variables could be used to describe 91.7% of the variation between active and inactive samples. The OPLS-DA score scatter plot revealed the clear separation of XvFr2, XvFr3, and XvFr4 from the rest of the bioactive fractions and extracts, which again suggests their chemical uniqueness. XvFr3 gave the most potent antibacterial activities against *S. aureus* and *E. coli.* From the loadings, five discriminating secondary metabolites were displayed and were assumed to be responsible for the observations in the score scatter plot. Interestingly, these five secondary metabolites were the same set of metabolites revealed as distinct in the PCA model based on the chemical profiles of all investigated samples. 

To further pinpoint the most significant metabolites responsible for the bioactivity, an S-plot was generated. In this study, the metabolite with the molecular weight of 196.11 Da (P_60, *m*/*z* 197.117) is situated on the tip of the “wing” of the active side of the S-plot far from the rest of the metabolites. This metabolite was dereplicated as 4-acetyl-2,4-octadienoic acid or methyl xylariate C. It has a high correlation to the model along with a high contribution to the variance making it the best prospective diagnostic metabolite for the bioactive *Xylaria venustula* samples. Through dereplication, this secondary metabolite was found to be common to *Xylaria* species. The ELF from which the respective metabolites were detected belongs to the same genus *Xylaria*, hence conducting further studies was not encouraged since this metabolite has already been explored. 

Four secondary metabolites situated next to P_60 were observed. Except for ophiocerin B (N_215), three of the discriminating metabolites (aplysinopsin (P_1587), methyl xylariate C (N_592), and piliformic acid (P_1117)) have been described in the literature for their bioactivities. Ophiocerin B was isolated from the fungus *Ophioceras venezuelense* and was described as not exhibiting antifungal (against *Candida albicans*) and antibacterial (against *S. aureus* and *E. coli*) activities in standard disk assays at 200 µg/disk [52]. Although ophiocerin B was found to be a distinct metabolite for the Xv fractions, it was not pinpointed among the bioactive metabolites. It should be noted, however, that the secondary metabolite N_592 is not identical to P_60 due to their different retention times. This may suggest that these two metabolites could be molecular isomers.

Visual interpretation of multivariate models, particularly the supervised models for OPLS-DA, could be potentially misleading unless accompanied by validating parameters [53]. In this study, the R^2^ value of the OPLS-DA model was close to 1; however, the Q^2^ value was low. This implies that the model explained the dataset well but has poor predictive power. Nevertheless, the CV-ANOVA *p*-values were identified. In this study, only methyl xylariate C (P_60) had a *p-*value of 0.05, which is significant. The rest of the discriminating metabolites had *p*-values greater than 0.05 but less than 0.2. It can be inferred that those secondary metabolites were detected by the model with approximately 15% of occurrence by chance. The *P*-values were at the margin of the significance level. However, the VIP scores of the pinpointed bioactivity were all greater than 5. Therefore, these compounds can still be targeted as potential diagnostic metabolites for “dual” bioactivity against *S. aureus* and *E. coli.*

The role of ELF on the lichen symbiosis has still not been fully investigated and remains unknown, although some assumptions have been made. For instance, the occurrence of ELF is presumed to play an important ecological role in lichens by assisting in lichen formation and growth, as well as by protecting the lichen host against insect herbivores through the production of bioactive compounds [54]. Our findings offer another interesting insight on the possible contribution of ELF to their lichen hosts. In this study, it was observed that different microorganisms were targeted by the metabolites produced by the lichens and their associated ELF. The lichen *Usnea* only inhibited the growth of *S. aureus,* as also observed in other studies [29,55]. In contrast, ELF inhibited both *S. aureus* and *E. coli*. We also observed bigger ZOIs from the ELF culture extracts than the lichen extracts, albeit the MIC values were lower for the lichen extracts. Interestingly, the differences in the bioactivities of ELF and their host lichens were further supported by the discovery of different set of metabolites produced by these organisms, as revealed by our metabolomics analyses. As such, we have come to believe that ELF contribute to the lichen association by producing different metabolites that may target other “competitor” microorganisms. While ELF’s motivation may be self-serving as they inhibit the growth of their potential competitors, their presence may have also protected the host lichens from other possible “harmful” or “invading” microorganisms. This has also been observed for fungal endophytes in plants, even though the plants and their associated fungal endophytes produce similar secondary metabolites [56]. Perhaps, by cultivating ELF in the presence of their host lichen thallus or lichen components, as those found in myco- and/or photobionts, may reveal similarities in metabolite production. These observations serve as an excellent stimulus to explore the metabolic pathways and metabolite production of both the lichen and its associated fungi. 

## 5. Conclusions

Among the lichen and ELF samples evaluated in this study, the ELF *X. venustula* showed potentially novel and bioactive antibacterial metabolites against *S. aureus*. To our knowledge, this is the first application of metabolomics and dereplication on the lichen *Usnea* from Malaysia and the Philippines and their associated ELF. Through this technology, the chemical natures of all lichen and ELF samples were revealed, which led to the discovery of the distinctiveness of the secondary metabolites produced by these organisms. Such a discovery also showed an interesting insight into the “protective role” of ELF towards their lichen hosts. Furthermore, the statistical models presented in this study also assisted in the identification of significant bioactive metabolites from ELF, particularly aplysinopsin (P_1587), piliformic acid (P_1117), and pestalopyrone A/tensyuic acid C/tensyuic acid D (P_1588), for future isolation work. The metabolomics approach conducted in this study allowed the rapid identification and targeting of possible diagnostic metabolites for bioactive natural products. 

## Figures and Tables

**Table 1:** The nine endolichenic fungi (ELF) species isolated from *Usnea* lichens collected from Bukit Larut, Malaysia (MY), and Sagada, Philippines (PH).

ELF Species	GenBank Accession Number	Lichen Host	Criterion for Selection	Reference ^1^
*Xylaria* sp.	MN071376	*U. bismolliuscula*	Abundance	Santiago et al. [15]
*Fusarium solani* (Mart.) Sacc.	MG881825	*U. pectinata*	Unique morphology	
*Pseudopestalotiopsis theae* (Sawada) Maharachch., K.D. Hyde and Crous	MG881833	*U. pectinata*		
*Xylariaceae* sp.	MN071378	*U. pectinata*		
*Astrocystis bambusae* (Henn.) Lćssře et Spooner	MH370741	*U. bismolliuscula*	Unique morphology	Santiago et al. [14]
*Annulohypoxylon albidiscum* J.F. Zhang, J.K. Liu, K.D. Hyde and Z.Y. Liu	MH370738	*U. pectinata*	Short incubation period (fast growth)	
*Daldinia eschscholtzii* (Ehrenb.) Rehm	MN071367	*U. pectinata*		
*Nemania bipapillata* (Berk. and M.A. Curtis) Pouzar	MN071354	*U. baileyi*	Abundance	
*Xylaria venustula* Sacc.	MH370742		Unique morphology	

^1^ Specific details on the collection sites and isolation and identification of these ELF species were separately reported in our other studies.

**Table 2:** Minimum inhibitory concentration (MIC) and minimum bactericidal concentration (MBC) values of the nine ELF crude extracts against *S. aureus* and *E. coli*.

ELF Species	Sample Code	Extract Yield ^1^ (g)	*S. aureus* ATCC 25923 (mg/mL)	*E. coli* ATCC 25922 (mg/mL)	References ^3^
*Xylaria* sp.	Xsp	3.873 (0.48%)	MIC and MBC: 10	MIC and MBC: 10	Santiago et al. [15]
*Fusarium solani*	Fs	3.196 (0.40%)	MIC and MBC: 10	No activity	
*Pseudopestalotiopsis theae*	Pt	1.4909 (0.18%)	MIC and MBC: 10	MIC and MBC: 10	
*Xylariaceae* sp. ^2^	Xcsp	2.357 (0.29%)	MIC: 1.25; MBC: 2.5	No activity	
*Astrocystis bambusae*	Ab	3.368 (0.42%)	MIC: 10; MBC: >10	No activity	Santiago et al. [14]
*Annulohypoxylon albidiscum*	Aa	3.630 (0.45%)	MIC: 2.5; MBC: 5	No activity	
*Daldinia eschscholtzii*	De	2.755 (0.34%)	MIC and MBC: 10	No activity	
*Nemania bipapillata*	Nb	2.300 (0.29%)	MIC and MBC: 10	MIC and MBC: 10	
*Xylaria venustula*	Xv	9.767 (1.22%)	MIC and MBC: 2.5	MIC and MBC: 5	

^1^ Crude extracts prepared from 800 g (5 × 160 g) rice culture media. ^2^ Discontinued due to unstable reproducibility to regrow the fungus during scale-up. ^3^ References where the MIC and MBC values were reported earlier.

**Table 3:** Discriminating secondary metabolites for ion peaks of *X. venustula* MH370742 putatively dereplicated from Dictionary of Natural Products (DNP) database.

MZMine ID ^1^	*m*/*z*	Retention Time	Molecular Weight	Molecular Formula	Dereplicated Identity	Reported Source ^2^	*p*-Value
P_60	197.117	11.55	196.11	C_11_H_16_O_3_	methyl xylariate C	*Xylaria* NCY2	0.05
P_1117	215.128	11.41	214.121	C_11_H_18_O_4_	piliformic acid	*Poronia piliformis, Xylaria longipes, X. polymorpha, X. hypoxylon, X. mali*	0.07
P_844	274.274	11.74	273.267	C_16_H_35_NO_2_	2-amino-1,3-hexadecanediol *N*-dodecyl-diethabolamine (DDE)	Various sponges marine-derived bacteria	0.09
N_592	195.103	13.29	196.11	C_11_H_16_O_3_	methyl xylariate C	*Xylaria* NCY2	0.11
P_1588	273.134	10.68	272.126	C_13_H_20_O_6_	pestalopyrone A tensyuic acid C tensyuic acid D	*Pestalotiopsis microspora* *Aspergillus niger* FKI-2342	0.11
P_1587	255.123	10.83	254.116	C_14_H_14_N_4_O	aplysinopsin	various marine invertebrates	0.11
N_215	131.071	7.25	132.079	C_6_H_12_O_3_	ophiocerin B	*Ophioceras venezuelense*	0.18

^1^ MZMine ID includes ionization polarity: P—positive mode, N—negative mode; ^2^ reported source: organism(s) from where the secondary metabolites were isolated according to DNP database.

## Data Availability

The data presented in this study are available in supplementary materials here.

## Supplementary Materials

The following are available online at https://www.mdpi.com/2079-7737/10/3/191/s1, Figure S1: PCA score plot of crude lichen and ELF extracts; Figure S2: Heatmap showing the comparative distribution of the metabolites ranked by t-tests in the respective classes (ELF: endolichenic fungi, UBS: *U. bismolliuscula*, UBY: *U. baileyi*, USP: *U. pectinata*, XSP: *Xylaria* sp., XV: *X. venustula*); Figure S3: **(a)** Heatmap and dendrogram of lichen and ELF extracts and fractions based on the top 25 metabolites (m/z-Rt), ranked according to their VIP scores. The most active fractions (ZOI ≥ 10 mm) are marked with an asterisk (*). Red box: lichen extracts and fractions; blue box: ELF extracts and fractions. **(b)** VIP scores of top 15 metabolites. The boxes on the right indicate the relative abundance of the metabolite in the respective classes. (ELF: endolichenic fungi, UBS: *U. bismolliuscula*, UBY: *U. baileyi*, USP: *U. pectinata*, XSP: *Xylaria* sp., XV: *X. venustula*); Figure S4: Multivariate analyses of 36 lichen and ELF crude extracts and selected fractions to predict the antibacterial metabolites in the *Xylaria* samples (Xsp and Xv). **(a)** Box-and-whisker plots of the predicted bioactive metabolites (*m*/*z*-Rt) indicating their relative occurrence in Xv fractions. **(b)** VIP scores bar plot for top 15 discriminating metabolites from both active and inactive classes. Those marked with an asterisk (*) are from the active Xv fractions, File S1: Supplementary Methods.
