# Supplementary material for: Biodiscovery of Potential Antibacterial Diagnostic Metabolites from the Endolichenic Fungus Xylaria venustula Using LC–MS-Based Metabolomics
Source: Biology (Basel). 2021 Mar 4;10(3):191. doi: 10.3390/biology10030191 (PMC8000601; doi:10.3390/biology10030191)
Supplement: Supplementary file 1 [file biology-10-00191-s001.zip › Supplementary File/Supplementary Materials.docx]

**Supplementary Materials: Figures**

**
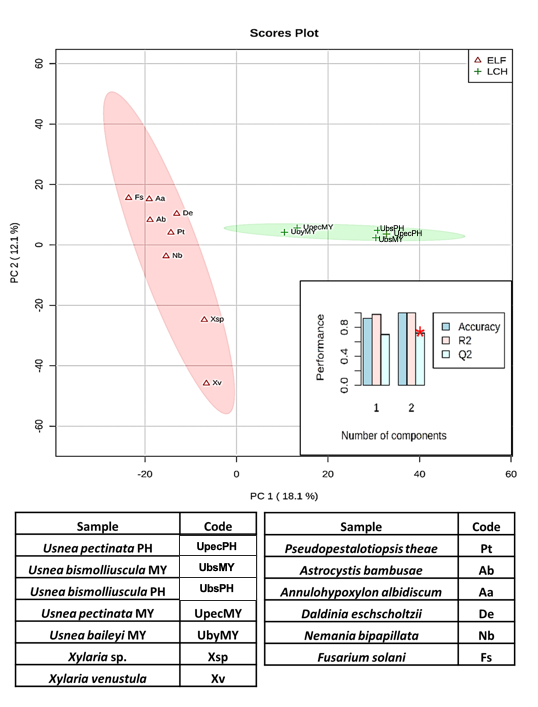
**

**Figure 1** PCA scores plot of crude lichen and ELF extracts

**
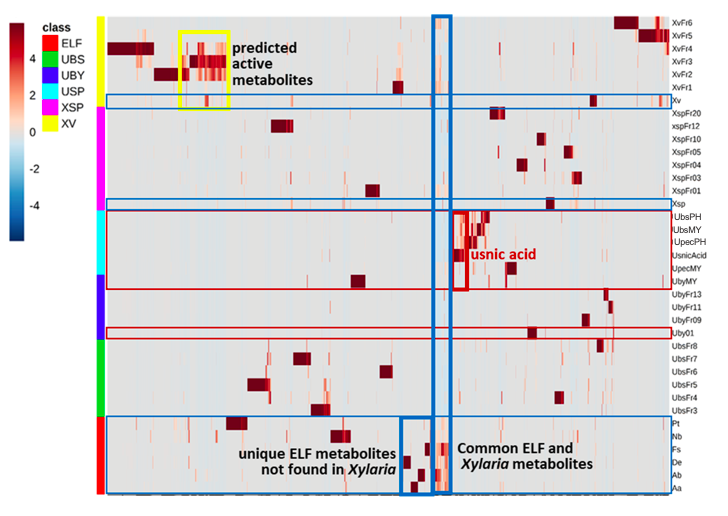
**

**Figure 2** Heatmap showing the comparative distribution of the metabolites ranked by t-tests in the respective classes (ELF: endolichenic fungi, UBS: *U. bismolliuscula,* UBY: *U. baileyi,* USP: *U. pectinata,* XSP: *Xylaria* sp., XV: *X. venustula*)

**
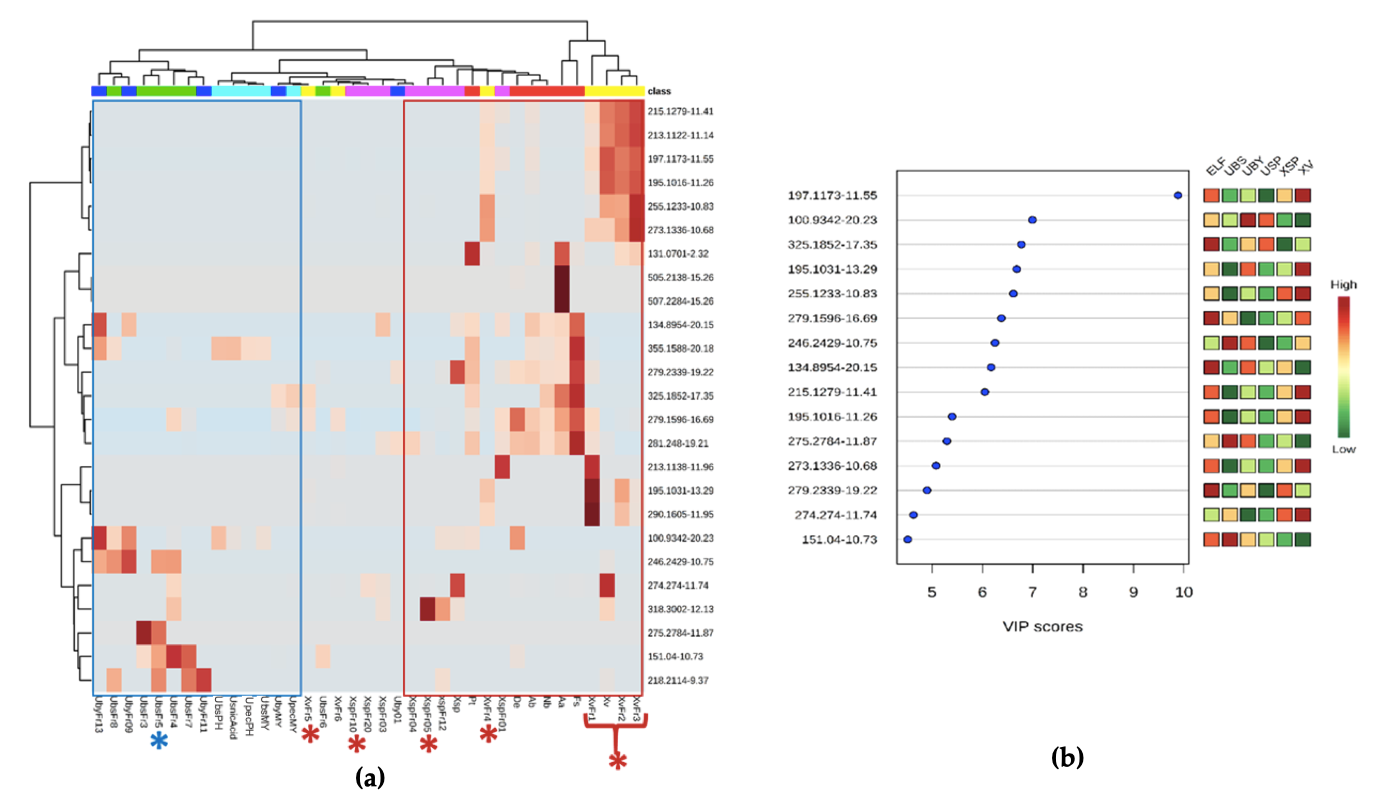
**

**Figure 3** **(a)** Heatmap and dendrogram of lichen and ELF extracts and fractions based on the top 25 metabolites (m/z-Rt) ranked according to their VIP scores. The most active fractions (ZOI > 10 mm) were marked with an asterisk (*). Red box: lichen extracts and fractions; Blue box: ELF extracts and fractions. **(b)** VIP scores of top 15 metabolites. The boxes on the right indicate the relative abundance of the metabolite in the respective classes. (ELF: endolichenic fungi, UBS: *U. bismolliuscula*, UBY: *U. baileyi*, USP: *U. pectinata*, XSP: *Xylaria* sp., XV: *X. venustula*)


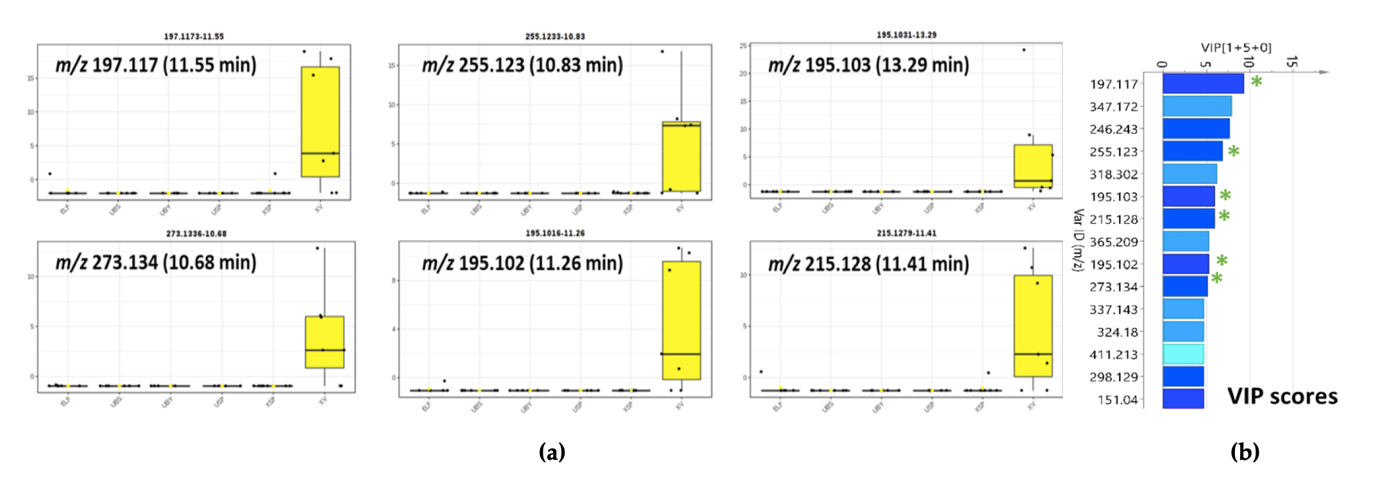


**Figure 4** Multivariate analyses of 36 lichen and ELF crude extracts and selected fractions to predict the antibacterial metabolites in the *Xylaria* samples (Xsp and Xv). **(a)** Box-and-whisker plots of the predicted bioactive metabolites (*m/z-*Rt) indicating their relative occurrence in Xv fractions. **(b)** VIP scores bar plot for top 15 discriminating metabolites from both active and inactive classes. Those marked with an asterisk (*) are from the active Xv fractions.
